# Expression of *TRIM56* gene in SARS-CoV-2 variants and its relationship with progression of COVID-19

**DOI:** 10.2217/fvl-2022-0210

**Published:** 2023-07-13

**Authors:** Rezvan Tavakoli, Pooneh Rahimi, Mojtaba Hamidi-Fard, Sana Eybpoosh, Delaram Doroud, Iraj Ahmadi, Enayat Anvari, Mohammadreza Aghasadeghi, Abolfazl Fateh

**Affiliations:** 1 Hepatitis & AIDS Department, Pasteur Institute of Iran, Tehran, Iran; 2 Viral Vaccine Research Center, Pasteur Institute of Iran, Tehran, Iran; 3 Department of Epidemiology & Biostatistics, Research Centre for Emerging & Reemerging Infectious Diseases, Pasteur Institute of Iran, Tehran, Iran; 4 Quality Control Department, Production & Research Complex, Pasteur Institute of Iran, Tehran, Iran; 5 Department of Physiology, School of Medicine, Ilam University of Medical Science, Ilam, Iran; 6 Department of Mycobacteriology & Pulmonary Research, Pasteur Institute of Iran, Tehran, Iran; 7 Microbiology Research Center (MRC), Pasteur Institute of Iran, Tehran, Iran

**Keywords:** C-reactive protein, coronavirus disease 2019, mild infection, SARS-CoV-2, severe infection, tripartite motif containing 56, viral RNA load

## Abstract

**Aim:**

The present study aimed to determine a correlation between differential *TRIM56* expression levels and severe infections of COVID-19 between the Alpha, Delta and Omicron BA.5 variants.

**Materials & methods:**

This study was performed on 330 COVID-19 patients, including 142 with severe and 188 with mild infections, as well as 160 healthy controls. The levels of *TRIM56* gene expression were determined using a qPCR.

**Results:**

*TRIM56* gene showed significantly lower mRNA expression in the severe and mild groups compared with healthy individuals. Our finding indicated the high and low reduction of *TRIM56* mRNA expression in Delta and Omicron BA.5 variant, respectively.

**Conclusion:**

Further research is needed to characterize the impact of TRIM proteins on the severity of COVID-19.

## Plain language summary

Scientists looked at a protein called TRIM that helps fight viruses to see if a specific TRIM protein, *TRIM56*, was linked to how poorly people became with COVID-19. The study looked at the blood samples of 330 patients and found that COVID-19 patients had less *TRIM56* than healthy people, especially those who were particularly ill.

## Tweetable abstract

#Tavakoli *et al.* discovered that the *TRIM56* gene, which plays a crucial role in the body's immune response to viruses, is likely associated with severe COVID-19 infections.

As the first line of defense against invading pathogens, the host innate immune system recognizes pathogen-associated molecular patterns (PAMPs) uisng pattern recognition receptors (PRRs) encoded by innate immune cells [1]. The toll-like receptors (TLRs) are PRRs that activate a wide variety of signaling pathways, inducing the transcription of interferon-I (IFN-I) and proinflammatory cytokines in cooperation with other PRRs [2,3]. Subsequently, IFNs can upregulate the expression of IFN-stimulated genes (ISGs) to stop the progression of infection. These processes are regulated by protein post-translational modifications including ubiquitination, methylation and phosphorylation to modulate the host's innate immune response to infection [4,5].

Pro- and anti-inflammatory responses during viral disease progression can result in apoptosis in endothelial and epithelial cells in the lung tissue due to a high and uncontrolled level secretion of cytokines like IL6, IL8 and TNF-a, known as a ‘cytokine storm’ [6,7]. Cytokine storm has been shown to be associated with severe COVID-19. This hyperinflammatory response plays a critical role in disease progression and clinical deterioration. Understanding the role of proinflammatory cytokines in cytokine storms and their association with severe COVID-19 is crucial for developing effective diagnostic and treatment strategies [8,9].

CRP is an acute-phase protein that is produced by the liver in response to inflammation and is considered a predictive indicator for severe inflammation in progressive respiratory failure by SARS-CoV2, MERS and SARS-CoV [10]. Previous studies have revealed that CRP levels were correlated with early lung tissue damage of COVID-19 pneumonia and could be associated with the severity of COVID-19 [11–13].

The tripartite motif (TRIM) protein family is the most extensive subfamily of the really interesting new genes (RING)-type E3 ubiquitin ligase family with E3 ubiquitin ligase activity in the N-terminal RING domain [14]. A wide variety of roles have been identified for TRIM protein in different cell processes. Some diseases such as systemic lupus erythematosus may develop by abnormality or impairment in the function of TRIM proteins. Recently, numerous studies have demonstrated that TRIM proteins have a vital role in antiviral innate immune responses [15,16]. They modulate the innate immune response by interfering with the post-translational modifications of cellular and/or viral proteins. In addition, the C-terminal of TRIM proteins interact with viral RNA or proteins and cellular proteins which may result in the suppression of viral replication. However, new research indicates that some viruses may usurp TRIM proteins to benefit their replication [17–19].

The *TRIM56* gene is a stimulator of interferon genes (STING) that enhances the antiviral potential of IFN-I and has been found to positively regulate the TLR3-mediated IFN pathway [20,21]. TLR3 and the RIG-I-like receptors both recognize the viral pathogen and trigger innate immune responses by producing interferon and proinflammatory mediators in the NF-κB pathway [17,22]. Understanding the important role of TRIM proteins and recognizing specific TRIM proteins in the regulation of the innate immune response needs further study. Moreover, TRIM proteins are able to target viral proteins for the ubiquitination process to disrupt virus replication [23]. The active region of the E3 enzyme is the RING structural domain of TRIM56, while the C-terminal region of TRIM56 mediates protein-protein or protein-RNA interactions between TRIM56 and cellular viral proteins/RNAs and can inhibit viral RNA replication [24,25]. TRIM56 has a direct role in restricting replicating coronaviruses and other positive single-stranded RNA viruses by blocking RNA replication, viral packaging or the release of the virus [26,27].

It has been found that some viral proteins can deactivate host restriction factors. This ability of viruses can be improved by the evolutionary arms race between host and virus, helping the virus escape from the host immune system with new variants resulting in a pandemic or endemic infectious disease, such as has been the case with SARS-CoV-2 and COVID-19 [28,29].

In view of this, we propose that *TRIM56* gene can be a possible regulator of infection with SARS-CoV-2 by moderating the innate immune response and inhibiting the replication of SARS-CoV-2. Therefore, we evaluated the mRNA level of *TRIM56* gene in our study groups of COVID-19 patients.

## Materials & methods

### Study population

This study included 300 patients with COVID-19 (severe and mild infection) and 160 uninfected subjects. Mild infection was defined as light clinical respiratory symptoms and fever without pneumonia, while severe infection was considered pneumonia, acute respiratory failure and an oxygen saturation of less than 92%. Patients with severe infections were hospitalized in the intensive care unit. Samples from uninfected controls were gathered at the Pasteur Institute of Iran (PII) before the COVID-19 pandemic.

The blood samples of COVID-19 patients were taken from November 2020 to February 2022, one week post the initiation of infection. During this time period, the most prevalent subtypes of the SARS-CoV-2 virus in Iran were the Alpha, Delta and Omicron BA.5 variants.

In this study, COVID-19 patients had no comorbidities or underlying disease such as diabetes, allergic diseases, chronic respiratory diseases, heart diseases or cancer.

The laboratory parameters, including lipid profiles, liver enzymes profile, fasting blood glucose (FBS), C-reactive protein (CRP), white blood cells (WBC), platelets, erythrocyte real-time PCR Ct values, creatinine, sedimentation rate (ESR), uric acid, and 25-hydroxyvitamin D, were extracted from the patients' records.

### RNA extraction

The messenger RNA (mRNA) of whole blood samples was extracted using RNA lysis buffer (Trizol: QIAGEN, USA) according to the supplier's protocol. The concentrations and purity of RNA were quantified by a Nanodrop spectrophotometer and RNA was stored at -80°C.

### Primer design & cDNA synthesis

The design stage of primers was performed by OligoAnalzer online tool in the exon-exon junction approach. The Refseq RNA sequences were taken from GenBank at National Center for Biotechnology Information (NCBI), and the predicted melting temperature was considered around 60°C. The primer sequences for *TRIM56* were 5′-CAGCTCTGGCTAGTTCTCAC-3′ and 5′-TCCTTCTAGTCTTCTGAGGC-3′. The housekeeping gene was and for hypoxanthine phosphoribosyltransferase 1 (*HPRT1*). A total of 1 μg of mRNA treated by the DNaseI enzyme (Thermo Fisher Scientific, USA) was reverse transcribed into cDNA according to the manufacturer's instructions (Yekta Tajhiz, Iran).

### Quantitative real-time PCR analysis for gene expression

The relative level of *TRIM56* was quantified by quantitative real-time PCR (qPCR) in two technical replicates (Corbett Rotor-Gene 6000, QIAGEN, USA) using SYBR green qPCR master mix (Ampliqon, Denmark). The cycling profile included 15 min at 95°C as the holding temperature followed by 15 sec at 95°C and 30 sec at 60°C for 40 cycles with the melting measure program at the end. The reference gene was selected out of four candidate's gene (*HPRT1*, *ACTB*, *B2M*, and *GRK6*). The *HPRT1* was used as the most stable housekeeping gene (M-value 1) in blood samples according to the ranking of a web tool named RefFinder [30], which uses different methods (Delta CT method, Normfinder, BestKeeper, and Genorm) to find most stable reference gene. For analyzing the gene mRNA expression levels, the comparative CT method (2^-ΔΔCT^ method) was used based on cycle threshold values.

### Statistical analysis

The qPCR results were analyzed statically using SPSS for Windows version 22.0. (SPSS, Inc., Chicago, IL, USA). The median and interquartile ranges (IQR) of non-parametric data were obtained. For assessing the normality of variables, the Shapiro-Wilk test was performed. Pearson's Chi-square test was used to analyze qualitative variables and Mann-Whitney test was used for quantitative variables. To assess the effect of the expression of *TRIM56* on the COVID-19 susceptibility, the curve-receiver operating characteristic analysis (ROC) and the area under curve (AUC) were performed. Spearman rank correlation analysis was used for determining the correlation between different variables. The statistical level of significance was considered p < 0.05.

## Results

### Baseline characteristics of COVID-19 patients

The comparative analysis of clinical features and laboratory results of COVID-19 patients is summarized in Table 1. The studied patients were separated into three groups: severe COVID-19 cases (n = 142), mild COVID-19 cases (n = 188) and healthy controls (n = 160). The mean ages of severe, mild and healthy subjects were 55.7 ± 11.7, 53.8 ± 11.8 and 54.9 ± 11.5 years, respectively. We observed that 174 (52.7%) COVID-19 patients and 83 (51.9%) healthy controls were male, respectively. High levels of ALT (p < 0.001), AST (p < 0.001), ALP (p < 0.001), CRP (p < 0.001), ESR (p < 0.001), FBS (p = 0.043) and creatinine (p < 0.001) and low levels of TG (p = 0.005), LDL (p < 0.001), uric acid (p < 0.001), real-time PCR Ct value (p = 0.008),and 25-hydroxyvitamin D (p < 0.001) were strongly linked with the severity of COVID-19.

**Table 1.** Comparison of laboratory parameters between mild and severe patients infected with COVID-19.

Variables	Mild patients (n = 188)	Severe patients (n = 142)	Control group (n = 160)	p-value
Mean age (years) ± SD	53.8 ± 11.8	55.7 ± 11.7	54.9 ± 11.5	0.755
Sex (male/female)	108/80 (57.4/42.6%)	66/76 (46.5/53.5%)	82/78 (51.2/48.8%)	0.171
ALT, IU/l (mean ± SD) (reference range: 5–40)	32.4 ± 24.5	46.1 ± 26.4	28.8 ± 19.3	<0.001^†^
AST, IU/l (mean ± SD) (reference range: 5–40)	29.2 ± 12.5	36.8 ± 14.9	27.6 ± 16.1	<0.001^†^
ALP, IU/l (mean ± SD) (reference range: up to 306)	124.7 ± 55.2	210.2 ± 68.7	145.8 ± 51.6	<0.001^†^
Cholesterol, mg/dl (mean ± SD) (reference range: 50–200)	127.8 ± 39.5	119.9 ± 50.8	151.9 ± 43.8	0.051
TG, mg/dl (mean ± SD) (reference range: 60–165)	135.4 ± 58.1	106.2 ± 35.2	148.2 ± 58.9	0.005^†^
LDL, mg/dl (mean ± SD) (reference range: up to 150)	127.9 ± 41.3	58.4 ± 18.1	112.8 ± 31.1	<0.001^†^
HDL, mg/dl (mean ± SD) (reference range: >40)	33.1 ± 11.1	31.7 ± 10.7	39.5 ± 13.6	0.358
WBC, 10^9^/l (mean ± SD) (reference range: 4000–10000)	7647.1 ± 2906.6	7691.8 ± 2810.5	6713.1 ± 2012.3	0.737
CRP, mg/l (mean ± SD) (reference range: <10 mg/l negative)	59.4 ± 21.5	75.5 ± 19.5	8.9 ± 2.2	<0.001^†^
ESR, mm/1st h (mean ± SD) (reference range: 0–15)	49.1 ± 16.5	74.3 ± 14.9	10.1 ± 4.2	<0.001^†^
FBS, mg/dl (mean ± SD) (reference range: 70–100)	106.2 ± 43.4	119.4 ± 51.3	98.3 ± 27.7	0.043^†^
Platelets × 1000/cumm (mean ± SD) (reference range: 140,000–400,000)	188 ± 85	194 ± 73	185 ± 81	0.190
T3, ng/dL (mean ± SD) (reference range: 2.3–4.2)	2.7 ± 0.6	2.4 ± 0.5	2.1 ± 1.1	0.596
T4, mcg/dl (mean ± SD) (reference range: 5.6–13.7)	10.0 ± 4.7	8.0 ± 4.1	11.4 ± 5.9	0.350
TSH, mu/l (mean ± SD) (reference range: 0.4–4.5)	3.3 ± 1.8	3.7 ± 1.9	2.8 ± 1.1	0.853
Uric acid, mg/dl (mean ± SD) (reference range: 3.6–6.8)	6.0 ± 1.4	3.7 ± 1.2	5.8 ± 1.6	<0.001^†^
Creatinine, mg/dl (mean ± SD) (reference range: 0.6–1.4)	0.7 ± 0.3	1.2 ± 0.8	0.9 ± 0.5	<0.001^†^
25-hydroxy vitamin D, ng/ml (mean ± SD) (sufficiency: 21–150)	33.9 ± 13.6	25.3 ± 9.7	42.2 ± 14.3	<0.001^†^
Real-time PCR Ct values	27.3 ± 8.5	11.9 ± 6.2	–	0.008^†^
SARS-CoV-2 variants				<0.001^†^
Alpha	56 (29.8%)	47 (33.1%)	–	
Delta	23 (12.2%)	78 (54.9%)	–	
Omicron BA.5	109 (58.0%)	17 (12.0%)	–	

† Statistically significant (<0.05).

ALP: Alkaline phosphatase; ALT: Alanine aminotransferase; AST: Aspartate aminotransferase; BUN: Blood urea nitrogen; Ct: Cycle threshold; ESR: Erythrocyte sedimentation rate; FBS: Fasting blood glucose; HDL: High-density lipoprotein; LDL: Low-density lipoprotein; SD: Standard deviation; TG: Triglyceride; T3: Triiodothyronine; T4: Thyroxine; TSH: Thyroid-stimulating hormone; WBC: White blood cell.

### Expression of *TRIM56* gene & COVID-19 progression

**Figure 1.**
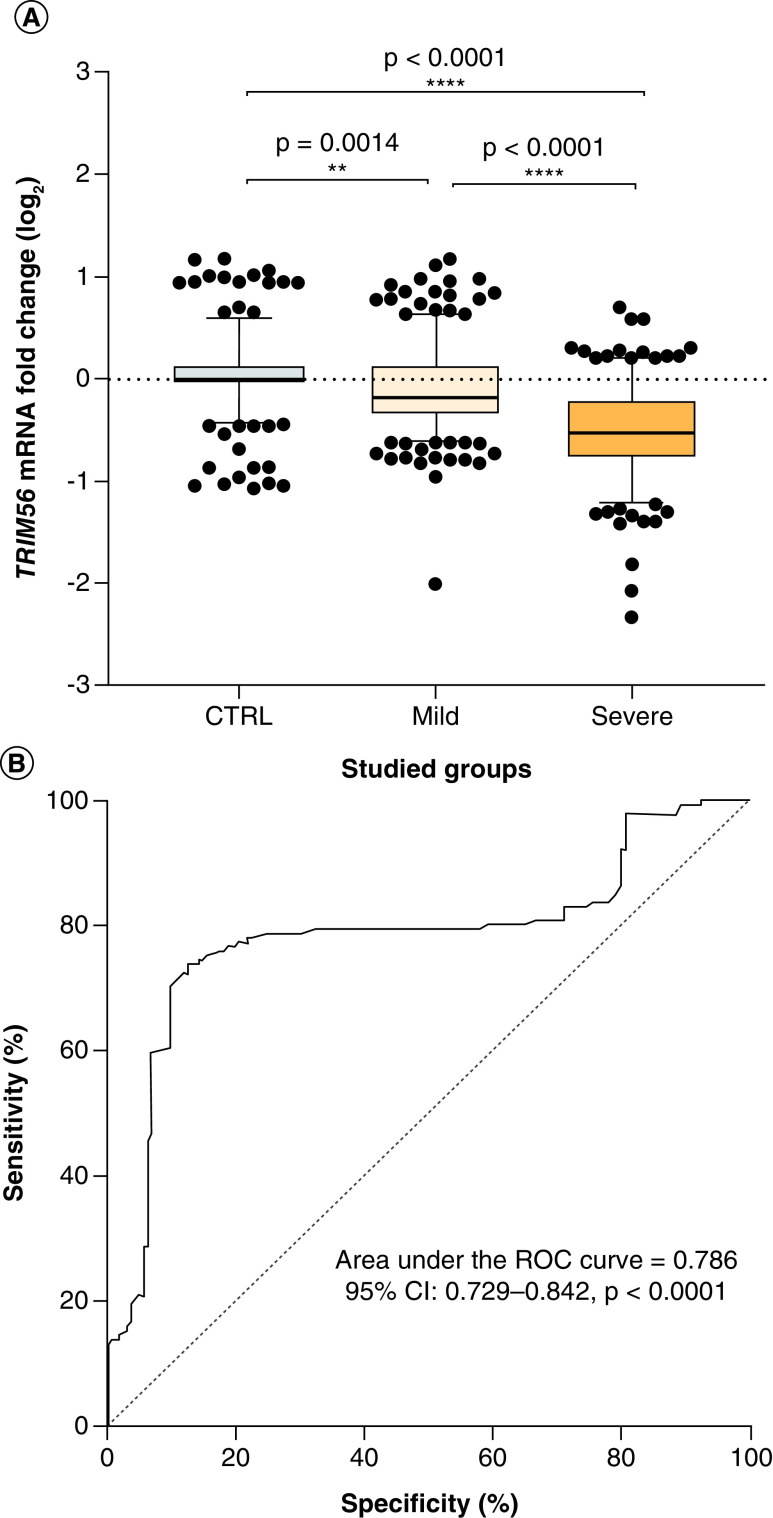
Expression of TRIM56 in studied subjects and receiver operating characteristic curve analyzing for predicting COVID-19 severity. **(A)** expression of TRIM56 in 490 whole blood samples from 3 groups: MILD and SEVERE COVID-19 patients (n = 330) and uninfected individuals as control group (n = 160). A horizontal line shows the median values with standard error of the mean bars. Median values and interquartile range (IQR) for TRIM56 CTRL: median 0.00, IQR -0.06, 0.14; MILD: median -0.18, IQR -0.35, 0.14; SEVERE: median: -0.53, IQR -0.78, -0.19. Statistical analysis: the Mann-Whitney test was used to compare the transcriptional levels of each group of patients with control group. **(B)** ROC curve with TRIM56 expression for predicting the severe COVID-19, Area under the ROC curve = 0.786, 95% CI: 0.729–0.842. ROC: Receiver operating characteristic curve.

Analysis of the expression levels of *TRIM56* mRNA in the severe group demonstrated a reduction in comparison to the uninfected control group (p < 0.0001). The mild group showed a reduction (median: -0.18) (p = 0.0014) versus the control group, but greater expression compared with the severe group (median: -0.53) (p < 0.0001) (Figure 1A). Additionally, the AUC-ROC value for *TRIM56* gene expression was 0.786, implying that the expression of this gene is related to the severity of COVID-19 (Figure 1B).

### Expression of *TRIM56* gene with different SARS-CoV-2 variants

**Figure 2.**
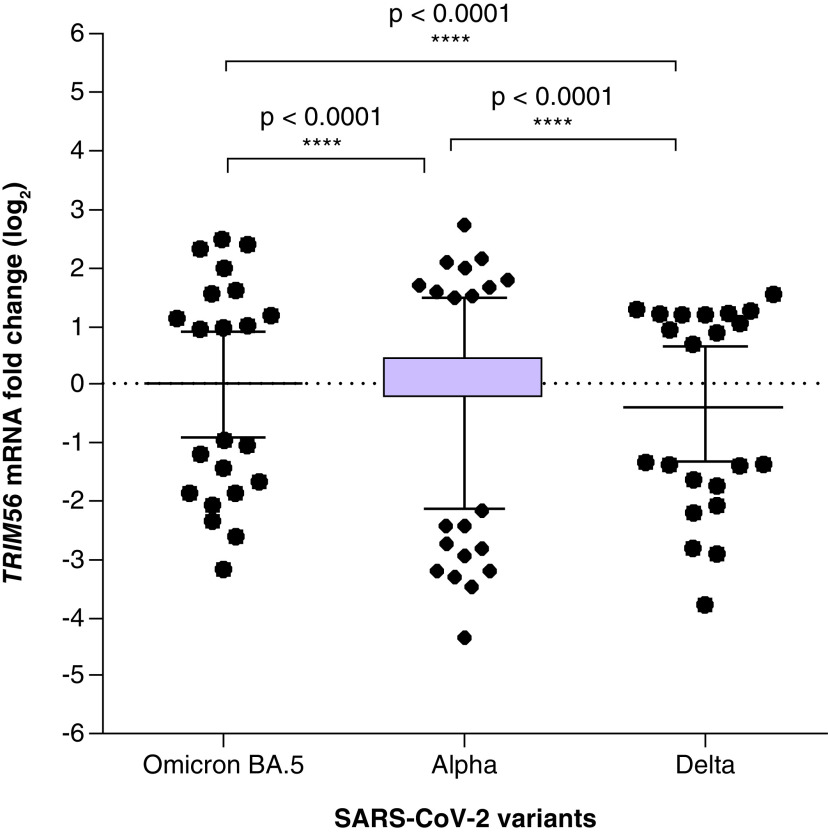
Expression of *TRIM56* in the Alpha, Delta and Omicron BA.5 variants. A horizontal line shows the median values with standard error of the mean error bars. Median values and interquartile range (interquartile range) for *TRIM56* gene.

We found higher reduction of *TRIM56* mRNA expression when patients were infected with the Delta versus the Omicron BA.5 variant (p < 0.0001). Infection with the Delta variant demonstrated lower levels of *TRIM56* mRNA expression when compared with both the Alpha and Omicron BA.5 variants (p < 0.0001). Infection with the Alpha variant was associated with a reduction in *TRIM56* mRNA expression (median: -0.15) (p < 0.0001) versus the Omicron BA.5 variant (Figure 2).

### Correlation between CRP value & the expression of *TRIM56* gene in COVID-19 patients

**Figure 3.**
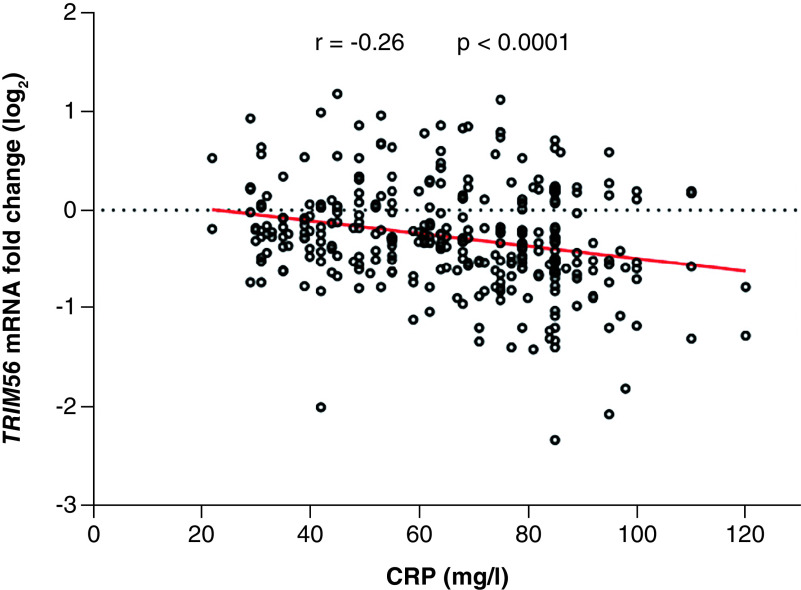
Correlations between CRP and *TRIM56* expression gene in patients with COVID-19.

Since previous studies reported that the CRP marker significantly correlated with the severity of COVID-19 as a predictor factor, we analyzed the relationship between *TRIM56* mRNA level and CRP value in all patients included in this study. We found a negative correlation, with high levels of CRP in patients related to lower levels of *TRIM56* expression (r = -0.26, 95% CI: -0.36 to -0/15, p < 0.0001) (Figure 3).

### Correlations between RNA viral load & the expression of *TRIM56* gene in COVID-19 patients

**Figure 4.**
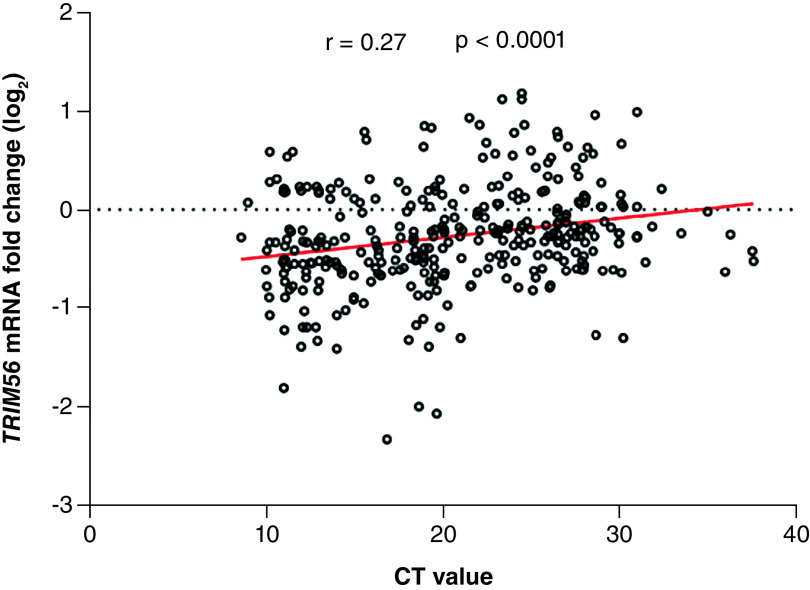
Correlations between cycle threshold value and *TRIM56* expression gene in patients with COVID-19.

We compared also Ct values and *TRIM56* mRNA levels of COVID-19 patients. Ct values were positively correlated with the expression of *TRIM56*. Lower Ct values indicating higher loads of viral RNA showed a significant relationship with lower levels of *TRIM56* expression (r = 0.27, 95% CI: 0.15 to 0.36, p < 0.0001) (Figure 4).

### Factors associated with the severity of COVID-19

The study conducted a multivariate logistic regression analysis to identify the factors that were associated with the severity of COVID-19 infection. The results indicated that several factors were significant predictors of severity. These factors included mean age (OR: 0.825, 95% CI: 0.792–0.923, p = 0.041), LDL (OR: 0.822, 95% CI: 0.792–0.923, p = 0.027), HDL (OR: 0.283, 95% CI: 0.170–0.523, p = 0.004), uric acid (OR: 1.163, 95%CI: 1.101–1.722, p < 0.001), creatinine (OR: 1.175, 95% CI: 1.122–1.523, p < 0.001), ALT (OR: 0.921, 95% CI: 0.873–0.983, p < 0.001), ESR (OR: 0.914, 95% CI: 0.899–0.992, p = 0.002), 25-hydroxyvitamin D (OR: 1.153, 95% CI: 1.091–1.410, p < 0.001), CRP (OR: 0.892, 95% CI: 0.810–0.992, p = 0.001) real-time PCR Ct values (OR: 2.215, 95% CI: 1.722–2.925, p < 0.001), and *TRIM56* gene expression (OR: 0.522, 95% CI: 0.470–0.822, p = 0.002), as shown in Table 2.

**Table 2.** Factors associated with severe patients infected with COVID-19.

Factors				
Baseline predictors	OR (95% CI)	p-value	aOR (95% CI)	p-value
Mean age ± SD	–	–	0.825 (0.792–0.923)	0.041^†^
LDL (mg/dl)	1.056 (1.038–1.074)	<0.001^†^	0.822 (0.701–0.908)	0.027^†^
HDL (mg/dl)	–	–	0.283 (0.170–0.523)	0.004^†^
Uric acid, mg/dl	2.863 (2.093–3.917)	<0.001^†^	1.163 (1.101–1.722)	<0.001^†^
Creatinine, mg/dl	0.006 (0.001–0.034)	<0.001^†^	1.175 (1.122–1.503)	<0.001^†^
ALT, IU/l	–	–	0.921 (0.873–0.983)	<0.001^†^
ESR, (mm/1st h)	–	–	0.914 (0.899–0.992)	0.002^†^
25-hydroxyvitamin D, (ng/ml)	1.070 (1.033–1.108)	<0.001^†^	1.153 (1.091–1.410)	<0.001^†^
CRP, (mg/l)	0.964 (0.947–0.981)	<0.001^†^	0.892 (0.810–0.992)	0.001^†^
Real-time PCR Ct values	1.175 (1.012–2.217)	0.021^†^	2.215 (1.722–2.925)	<0.001^†^
*TRIM56* gene expression	0.801 (0.727–0.882)	<0.001^†^	0.522 (0.470–0.822)	0.002^†^

† Statistically significant (<0.05).

ALT: Alanine aminotransferase; aOR: Adjusted odds ratio; CI: Confidence interval; Ct: Cycle threshold; ESR: Erythrocyte sedimentation rate; HDL: High-density lipoprotein; LDL: Low-density lipoprotein; SD: Standard deviation.

## Discussion

We evaluated whole blood samples of COVID-19 patients to assess the correlation between the severity of SARS-CoV-2 and *TRIM56* expression. We also wanted to find the relationship between *TRIM56* expression and two clinical laboratory factors, including the CRP marker and Ct value of the qPCR test.

A significant correlation between increased CRP value and decreased *TRIM56* gene was identified in this study. Alteration in the expression of cytokines and immune mediators in SARS-CoV-2 infection results in cytokine storm and systematic inflammation in severe cases, which is related to high mortality in COVID-19 [31–33]. Cytokines such as IL-6, IL8 and TNF-α induce the production of CRP by hepatocytes. CRP can activate the complement system to remove necrotic cells and various pathogens [11]. Recent studies have reported that CRP is a strong biomarker relating to the severity and mortality rate of COVID-19 infection [34,35]. Specifically, CRP levels have been shown to be correlated with adverse COVID-19 outcomes. Hence, monitoring CRP levels can provide valuable insights into disease progression, allowing clinicians to make informed therapeutic decisions [8]. Plus, elevated levels of CRP were found in up to 86% of severe COVID-19 cases in some studies, with levels typically ranging from 20 to 50 mg/l on average [36–38]. On the other hand, TRIM56 is known as an IFN-inducible gene and a positive regulator of TLR3 which can induce interferon and proinflammatory mediators produced during viral infection [16,20]. Reduction of the *TRIM56* gene in viral infection may lead to the unregulated generation of IFN and cytokines.

The expression of *TRIM56* was expected to be greater in SARS-CoV-2 infection according to previous studies [39]. In contrast, our results showed a notable reduction in the expression of *TRIM56* mRNA in severe patients compared with healthy controls and patients with mild COVID-19. Wang *et al.* reported in 2011 that *TRIM56* has an innate antiviral effect against bovine viral diarrhea virus (BVDV), an RNA virus with positive-strand, in an *in vitro* study. They showed that *TRIM56* interacted with the BVDV N-terminal protease via its C-terminal and blocked the function of this protein in BVDV with interferon regulatory factor 3 (IRF3). However, a large deletion in the N-terminal of TRIM56 had an important effect on the folding of the protein and its interaction with the protease of BVDV [40]. Moreover, TRIM56 as an antiviral factor in host cells is able to restrict the replication of some viruses from the Flaviviridae family like dengue virus-2 and yellow fever virus [27]. Wang *et al.* also confirmed that a decline in *TRIM56* expression level leads to the replication of BVDV [40]. The antiviral effects of TRIM56 are virus-dependent, because augmenting the expression of *TRIM56* in human hepatoma (Huh7) cells did not restrict the repetition of the hepatitis C virus (HCV) [41]. Furthermore, in RNA viruses with negative strands, such as influenza A virus (IAV) and Infectious Bronchitis Virus (IBV), the upregulation of *TRIM56* inhibited viral replication by blocking viral RNA synthesis [26]. Plus, TRIM56 has a direct restriction role in the replication of coronaviruses and other positive-sense single-stranded RNA viruses [21]. It has been demonstrated that TRIM56 can target viral proteins that control the packaging stage and virus release to prevent replication of HCoV-OC43 [27]. In addition, in agreement with our result, recent research that analyzed the transcriptional patterns of different cells using RNA-seq data after infection by a handful of viruses including IAV, SARS-CoV-2 and MERS-CoV showed a significant reduction in *TRIM56* for IAV and SRAS-CoV-2 in A549 cells. Analysis of lung tissue samples of COVID-19 patients also reported a decrease in *TRIM56*, though this was not significant (*P* 0.09) [42]. It is possible that *TRIM56* gene has some polymorphisms in regulatory sequences that affect its expression in COVID-19 patients, or that SARS-CoV-2 has a genetic mutation that can facilitate the inhibition or reduction of the expression of *TRIM56* gene. The impact and antiviral mechanisms of *TRIM56* gene in COVID-19 need further investigation.

We also assessed the correlation between the Ct value of the qPCR test and the mRNA expression level of *TRIM56* in patients. Ct value of the qPCR test is considered a simple clinical marker of COVID-19 infection as it indicates viral RNA load [43,44]. There are numerous factors that can lead to false results in these tests, however, such that many researchers avoid accepting Ct value as a predictive biomarker for the severity of infection [45]. In this study, we considered several factors to gain reliable Ct values from the qPCR test like sampling time, technique, site and stage of disease when obtaining samples. Our findings demonstrated a considerable correlation between high viral loads and the low level of *TRIM56* mRNA in patients. We also showed a significant reduction of *TRIM56* gene expression in severely ill patients. TRIM56 has a restrictive role in the replication of RNA viruses like HCoV-OC43, so it is likely that TRIM56 similarly interacts with SARS-CoV-2 and as a result, the reduction of *TRIM56* observed in patients with severe COVID-19 may be associated with the superior viral load in these patients.

The multivariate logistic regression analysis of results showed that several factors were significant predictors of the severity of COVID-19. These factors include mean age, LDL, HDL, uric acid, creatinine, ALT, ESR, 25-hydroxyvitamin D, CRP, real-time PCR Ct values and *TRIM56* gene expression. Higher levels of CRP, lower levels of *TRIM56* gene expression and lower Ct values were associated with an increased risk of severe disease. Studies have shown that COVID-19 severity may be linked to elevated liver enzyme levels, which may indicate liver injury. This injury is characterized by abnormal liver enzyme levels and slightly increased bilirubin concentrations [46,47]. Although the precise cause of liver injury in COVID-19 patients is not yet known, various factors such as direct virus-induced cytopathogenic effects, worsening of preexisting liver disease, hypoxia, drug-induced effects and an excessive inflammatory response are thought to contribute to it [48]. Previously it has been found that high levels of plasma lipids or hyperlipidemia are commonly associated with other underlying health conditions such as obesity, diabetes mellitus, or cardiovascular diseases, which are known to impact the prognosis of COVID-19 patients [49]. Patients who have been previously treated with statins, the most commonly prescribed lipid-lowering medication, also have a higher chance of survival, indicating that statin use might have a role in predicting augmented cardiometabolic risk in COVID-19 [50]. Interestingly, low cholesterol levels were found in COVID-19 patients with poor prognoses, which could be attributed to malnutrition or the severity of inflammation in COVID-19 patients with the poorest outcomes [51]. According to our research, there is further evidence to suggest that a lack of 25-hydroxyvitamin D may increase the risk of contracting severe COVID-19. Recent studies have uncovered a relationship between SARS-CoV-2 and the ACE2, whereby the virus can significantly reduce *ACE2* expression, thereby contributing to the severity of COVID-19 [52,53]. 25-hydroxyvitamin D is known to affect the renin-angiotensin system, which plays a role in regulating blood pressure and fluid balance in the body, and also stimulates the expression of *ACE2* [54]. Because high levels of *ACE2* expression have been linked to more severe COVID-19, it remains unclear how much vitamin D may actually benefit those with the disease. Further studies are needed to develop effective treatment strategies for COVID-19 across different levels of severity [55]. Many patients also had low levels of serum uric acid, which was associated with increased severity of COVID-19, leading to the need for invasive mechanical ventilation. The reasons why hypouricemia is linked with the development of severe COVID-19 disease are not entirely clear, and several possibilities exist. One possibility is that uric acid is an important antioxidant and low levels of this molecule could result in weakened defense against oxidative stress, contributing to the severity of the disease [56]. Another possibility is that low blood uric acid levels may worsen the cytokine storm in COVID-19, as uric acid has been shown to play a role in modulating the immune response [57]. Finally, recent research suggests that severe hypouricemia can cause endothelial dysfunction, lower blood pressure, reduce myeloperoxidase activity and promote lipid peroxidation in healthy individuals, which could worsen the outcome of COVID-19 patients with low uric acid levels [57].

In agreement with our previous studies [15,18,19], these results suggest that the levels of several biomarkers and gene expression may be useful predictors of the severity of COVID-19. Further studies are needed to confirm these findings and determine the underlying mechanisms of these associations.

## Conclusion

Few researchers have reported on the activity of TRIM proteins during SARS-CoV-2 infection. Our study provides a clue to the TRIM proteins' role in COVID-19 and focuses on expression of the *TRIM56* gene in blood samples. Large-scale research on different aspects are recommended to confirm the findings mentioned above. It's important to note that these findings were observed in the specific population studied and may not be generalizable to other populations.

**Summary points**

- The expression level of *TRIM56* mRNA in the severe group showed a significant reduction in comparison with the uninfected control group.
- The mild group represented a meaningful decrease versus the control group and had upregulation compared with the severe group.
- The area under curve-receiver operating characteristic analysis value for *TRIM56* gene expression was 0.786, implying that the expression of this gene is frequently required for the severity of COVID-19.
- Our finding indicated the high and low reduction of *TRIM56* mRNA expression was shown in Alpha, Delta and Omicron BA.5 variants, respectively.
- Expression mRNA level of *TRIM56* in patients had a meaningful correlation with CRP value and viral RNA load with r = -0.26 and r = 0.27 respectively.
